# Proteomics Insights Into Lysosome Biogenesis and Maturation

**DOI:** 10.1002/pmic.70058

**Published:** 2025-10-15

**Authors:** Katharina Hirn, Sofía Fajardo‐Callejón, Dominic Winter

**Affiliations:** ^1^ Department Metabolism Senescence and Autophagy Research Center One Health Ruhr Medical Faculty University Alliance Ruhr, University Hospital Essen University of Duisburg‐Essen Essen Germany; ^2^ Institute for Biochemistry and Molecular Biology Medical Faculty Rheinische Friedrich‐Wilhelms‐University of Bonn Bonn Germany

**Keywords:** biogenesis, lysosomal proteins, lysosome, mass spectrometry, maturation, protein trafficking, proteomics, vesicular transport

## Abstract

Lysosomes constitute the main degradative organelle of most eukaryotic cells and are capable of breaking down a wide spectrum of biomolecules, including proteins, lipids, glycans, and DNA/RNA. They play crucial roles in the regulation of cellular homeostasis, acting as metabolic signaling centers for the correlation of nutrient availability and biosynthetic processes. The lysosome's importance is highlighted by several human diseases associated with its dysfunction, including both early‐ and late‐onset conditions, dependent on the level of functional impairment. Lysosomal biogenesis presents a multi‐step process consisting of various delivery routes for its individual constituents, enabling strict activity control of the currently known ∼60 lysosomal hydrolases to prevent cellular self‐digestion and proper assembly of the lysosomal membrane. In this review, we recapitulate the contribution of mass spectrometry (MS)‐based proteomics to the characterization of lysosomal biogenesis in the last two decades. The enrichment and proteomic analysis of lysosomes and lysosomal proteins played an invaluable role for the investigation of lysosomes, encompassing the control of lysosomal gene expression, the characterization of sorting/trafficking processes, and the assignment of lysosomal proteins. This has resulted so far in the definition of ∼350 proteins which have been identified to be located in/at lysosomes or are of crucial importance for their function.

## 1 Introduction

Lysosomes are highly dynamic organelles at the center of cellular metabolism and are present in the vast majority of eukaryotic cells [1]. Characterized by an acidic pH (4.5–5.5), they are responsible for the breakdown and recycling of all major classes of biological macromolecules, that is, proteins, lipids, sugars, and nucleic acids. The resulting basic building blocks are transported to the cytoplasm, providing a continuous turnover of both intra‐ and extracellular components [2]. Lysosomes receive their substrates from multiple routes that mostly depend on vesicular transport [1, 3]. Endocytosis and phagocytosis deliver extracellular material such as cell surface proteins or receptor‐bound low‐density lipoproteins [4] and apoptotic cell debris or pathogens [5, 6], respectively. Autophagy, on the other hand, transports intracellular components through various routes to lysosomes, playing a crucial role in the removal of cytosolic content such as protein aggregates and organelles [7, 8]. While macroautophagy refers to the degradation of cellular material delivered to the lysosome through autophagosomes [7], microautophagy is the direct uptake of cytoplasmic material by the lysosome [9]. Also, single proteins can be imported into the lysosome by chaperone‐mediated autophagy, enabling their selective degradation [10].

Aside from their degradative functions, lysosomes are by now well‐established as signaling hubs with critical roles in nutrient sensing, gene regulation, protein synthesis, and cell growth [11]. In this context, the lysosomal surface acts as a platform for the activation of the mechanistic target of rapamycin complex 1 (mTORC1) and the 5’‐AMP activated protein kinase (AMPK), responding to both lysosomal and cellular levels of nutrients through various sensors [12, 13]. The degradative function of lysosomes is closely connected to their cytoplasmic positioning, which is dependent on bidirectional movement along microtubule tracks and facilitated by a variety of motor protein complexes [14, 15]. These changes in position also allow lysosomes to interact with other organelles for the exchange of metabolites [16].

The crucial role of lysosomal function in cell homeostasis is highlighted by the consequences of lysosomal dysfunction, which is known as the underlying cause of various human diseases. Loss of function mutations in lysosomal hydrolases, as well as several other lysosome‐related proteins, lead to the accumulation of lysosomal substrates and, consequentially, impairment of lysosomal activity. This results in so‐called lysosomal storage disorders (LSDs), a class of ∼70 rare inherited diseases. LSDs are frequently lethal and currently the most prevalent known cause for neurodegeneration in children. Importantly, therapeutic options for the treatment of LSDs are very limited [17]. A reduction of lysosomal function has been further implicated for a variety of more common diseases in recent years, such as neurodegenerative diseases, diabetes mellitus, and atherosclerosis [18, 19, 20]. Notably, increased lysosomal function is also known to play an important role in multiple types of cancers, where it enables, among others, metabolic adaptation and tissue invasion [11]. So far, more than 1000 studies identified changes in lysosomal genes, transcripts, or proteins in human patient samples from more than 250 pathological conditions [21], further highlighting the important function of the lysosome in human disease.

**FIGURE 1:**
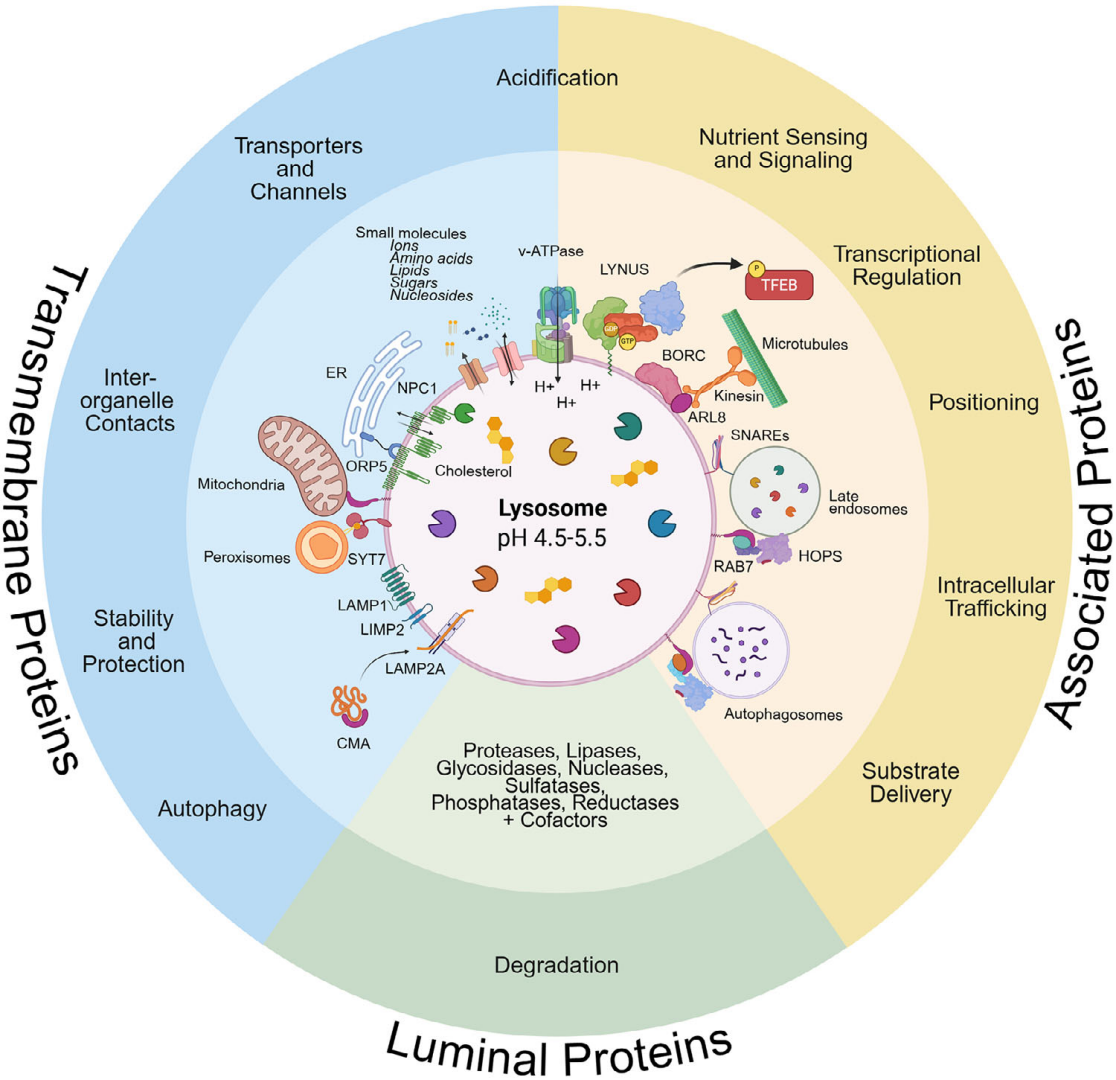
Composition and functions of the lysosomal proteome. Lysosomal proteins can be grouped into luminal, integral transmembrane, and membrane‐associated proteins. Approximately 60 hydrolases mediate the degradation of substrates in the lysosome. Lysosomal membrane proteins include highly glycosylated integral membrane proteins (LIMPs) and lysosome‐associated membrane proteins (LAMPs) that form a protective glycocalyx at the luminal side of the lysosomal membrane. LAMP2 further participates in chaperone‐mediated autophagy (CMA). Other important proteins and complexes of the lysosomal membrane include the vacuolar H^+^‐ATPase complex, which is responsible for acidification of the lysosomal lumen, transporters/exchangers for small molecules, and docking machineries for inter‐organelle contact sites. Several cytosolic lysosome‐associated proteins dynamically interact with the lysosomal membrane, such as members of the mechanistic target of rapamycin complex 1 (mTORC1), which participate in cellular signaling, or the transcription factor EB (TFEB), a regulator of lysosome biogenesis. The multi‐subunit BLOC1‐related complex (BORC) and the homotypic fusion and vacuole protein sorting (HOPS) complex are crucial for lysosomal positioning and fusion with other vesicles, such as endosomes and autophagosomes.

The lysosome's diverse functions are carried out by the lysosomal proteome, which encompasses a variety of luminal hydrolases and their cofactors, membrane‐localized exchangers/transporters and structural proteins, as well as numerous lysosome‐associated proteins (Figure 1) [1, 2, 22]. To date, more than 900 proteins were flagged as “lysosomal” in gene ontology (GO) databases and/or UniProt, respectively, of which 348 have been validated to be lysosome‐related in low‐throughput studies (Figure 1, Table S1).

Lysosomal function relies on a continuous delivery of newly synthetized lysosomal proteins, as its high hydrolytic activity results in a steady turnover for many of them [3]. The delivery of lysosomal proteins is based on two sorting mechanisms: transmembrane lysosomal proteins contain cytosolic sorting motifs that are recognized by adaptor complexes in the *trans*‑Golgi network (TGN), whereas soluble hydrolases acquire a mannose‑6‑phosphate (M6P) tag in the *cis*‑Golgi and are then captured by M6P receptors (MPRs) in the TGN for packaging into clathrin‑coated vesicles [23].

Several mass spectrometry (MS)‐based experimental strategies have been developed to investigate the dynamics of the lysosomal proteome, and hence its biogenesis, that can be broadly classified in the enrichment of intact organelles and of specific proteins. While these approaches initially heavily relied on stable isotope labeling experiments, gel‐based approaches, and data‐dependent acquisition (DDA) strategies [24, 25], recent studies often utilize label‐free quantification by data‐independent acquisition (DIA) [26, 27, 28].

### 1.1 Enrichment of Intact Lysosomes

After the selective disruption of the plasma membrane and the depletion of intact cells and debris by centrifugation, lysosomes can be enriched from the postnuclear supernatant. Currently, three strategies are widely used, which can be categorized into methods based on differential centrifugation, superparamagnetic iron oxide nanoparticles (SPIONs), and immunoprecipitation of membrane proteins (Lyso‐IP).

**FIGURE 2:**
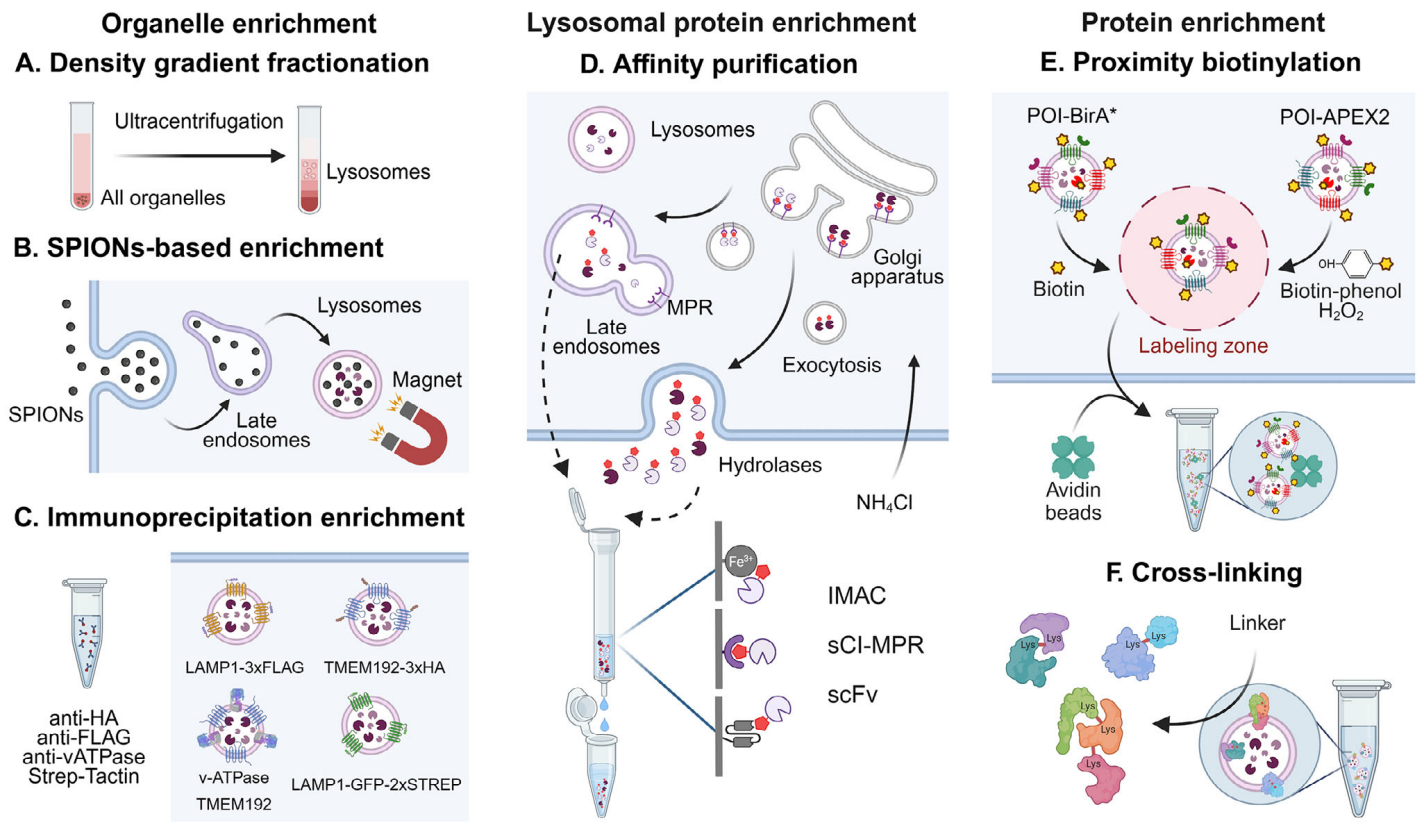
Strategies for the enrichment of lysosomes and lysosomal proteins. (A) Cells or tissue are homogenized and lysosomes are enriched based on their sedimentation coefficient in ultracentrifugation experiments. Further selectivity can be achieved by density gradients using specific matrices. (B) Superparamagnetic iron oxide nanoparticles (SPIONs) reach the lysosomes of cells grown in culture through endocytosis. Using a strong magnetic field, intact SPIONs‐containing lysosomes can be enriched. (C) Immunoprecipitation of intact lysosomes is performed using immobilized antibodies against tagged or endogenous lysosomal membrane proteins. (D) Lysosomal hydrolases are modified in the Golgi apparatus with a mannose‐6‐phosphate (M6P) tag for their subsequent delivery to lysosomes through trafficking vesicles or the secretory pathway. Using immobilized metal ion affinity chromatography (IMAC), immobilized M6P receptor fragments (sCI‐MPR), or single‐chain antibodies (scFv), M6P‐tagged proteins can be enriched. Treatment with NH_4_Cl triggers the secretion of M6P‐containing hydrolases from the Golgi. (E) For proximity biotinylation of interacting proteins, lysosomal membrane proteins are tagged with either BirA*, TurboID, or APEX. Upon the addition of biotin or biotin‐phenol/H_2_O_2_, respectively, proximal proteins are biotinylated and can be enriched using avidin. (F) The cross‐linking of enriched lysosomes results in the formation of covalent bonds between interacting proteins whose amino acid side chains are located within a certain distance, enabling the identification of protein‐protein interactions.

Differential centrifugation utilizes the size and sedimentation coefficient of lysosomes, resulting in specific migration behaviors in density gradient centrifugation experiments [29] (Figure 2A). The most commonly used matrix in this context is sucrose [30]. However, especially as mitochondria have a similar sedimentation coefficient as lysosomes in such experiments, multiple other matrices have been introduced, including metrizamide [31], Nycodenz [32], Percoll [33], and iodixanol [34], which enable to yield higher lysosomal purity. Furthermore, several strategies for the loading of lysosomes with reagents that alter their density, and hence enable their separation from other organelles, have been developed [35, 36]. For differential centrifugation approaches, lysosomal proteins can also be identified in experiments covering the whole proteome using protein correlation profiling [37]. This approach is based on the assumption that proteins from the same organelle will be co‐detected in individual fractions of a centrifugation experiment, as they co‐migrate due to their common location at the organelle of interest. Therefore, they can be assigned to specific (sub‐) cellular compartments based on protein abundance profiles derived from quantitative proteomics experiments [38].

The use of SPIONs for cells growing in culture is based on unspecific fluid‐phase endocytosis and hence their uptake by early endosomes. Subsequently, the nanoparticles reach lysosomes through the endocytic route, and cell type‐specific adjusted pulse‐chase conditions enable (almost) exclusive localization to terminal lysosomes, which can then be enriched by a strong magnetic field [26, 39] (Figure 2B). Variations of SPIONs have been developed, involving different surface chemistry, polarity, and charge, resulting in different uptake kinetics by cells [40, 41].

Lyso‐IP is based on the immunoprecipitation (IP) of intact lysosomes using immobilized antibodies against lysosomal membrane proteins (Figure 2C). This typically requires the overexpression of tagged membrane proteins, for which currently a 3xHA‐tagged version of the lysosomal membrane protein TMEM192 is used most commonly [42], but also mRFP‐2xFLAG‐ or 3xFLAG‐tagged versions of LAMP1 [12, 43] have been utilized. Similarly, the enrichment of lysosomes based on the IP of endogenous membrane proteins has been reported, utilizing antibodies against the cytosolic A/B subunits of the vacuolar H^+^‐ATPase (v‐ATPase) complex [44], and more recently, TMEM192 (tagless Lyso‐IP) [45].

While all of these approaches have certain advantages and disadvantages, such as yield/purity [39], applicability to animal models [46, 47], or effects on the lysosomal proteome [26, 48], they significantly contributed to our understanding of lysosomal biology. In general, differential centrifugation presents the most universal approach, as it can be applied to virtually any sample type as long as intact lysosomes can be released by selective rupture of the plasma membrane. This is also the case for tagless Lyso‐IP strategies targeting endogenous proteins. Their efficiency, however, has not been tested in many settings yet. Conversely, the use of SPIONs is restricted to cells grown in culture which actively endocytose extracellular material, and Lyso‐IP to the overexpression of tagged proteins. While the latter is one of the most frequently utilized strategies, it's limited to cell lines or animal models that allow for genetic manipulation.

### 1.2 Characterization of Lysosomal Proteins by Affinity Enrichment or Targeted MS

In addition to intact organelles, lysosomal luminal proteins can be specifically enriched from whole cell lysates by affinity purification through their M6P tag. For this, multiple strategies have been established, such as enrichment by immobilized soluble fragments of the M6P receptor or an M6P‐specific single‐chain antibody, as well as immobilized metal ion affinity chromatography (IMAC, Figure 2D) [49, 50, 51]. These strategies are well‐suited to investigate lysosomal biogenesis, as the majority of lysosomal luminal proteins are M6P‐modified. They are not applicable, however, to the investigation of mature lysosomes, as the M6P tag is instantly removed by the lysosomal phosphatase ACP2 and ACP5 after a protein reaches the lysosomal lumen.

Furthermore, proteins residing in, or otherwise associated with, the lysosomal membrane can further be labeled by proximity biotinylation, enriched by avidin, and identified using LC‐MS/MS [52]. Proximity biotinylation approaches can be used for lysosomal proteins at any stage of lysosomal biogenesis. For such analyses, either BirA*/TurboID [53, 54] or APEX/APEX2 [55, 56] can be fused to a lysosomal membrane protein of interest, enabling the biotinylation of proteins interacting with the lysosomal membrane in situ. Previously, LAMP1‐APEX2 [57, 58] or BirA* coupled to LAMTOR1, BORCS6, STX7, RAB9A, LAMP1, LAMP2, and LAMP3 have been utilized in such experiments [59]. More recently, the biotinylation of endogenous lysosomal proteins in PFA‐fixed mouse brain slices, through antibody recognition of horseradish peroxidase fusion protein (Lyso‐BAR), was reported [60].

Enzyme‐based proximity biotinylation approaches in living cells are limited to the cytosolic face of lysosomes, as BirA*/APEX would be degraded in the lysosomal lumen. To address this issue, several photolabeling strategies were recently developed, which further allow for the labeling of luminal proteins within terminal lysosomes [61, 62] (Figure 2E). Finally, crosslinking mass spectrometry (XL‐MS), which is based on the covalent linkage of two functional groups in adjacent proteins, such as primary amines in lysine residues, has been utilized to investigate lysosomal protein‐protein interaction. Through labeling of both intact and disrupted lysosomes, this approach covered both lysosomal luminal and surface proteins [27] (Figure 2F).

Besides the spatial information, which is gained through the enrichment of lysosomal proteins or intact organelles, an essential factor is the reduction of sample complexity and hence the increase of signal intensity of lysosomal proteins. For unbiased MS analyses, the quantification performance for lysosomal proteins in whole cell or tissue lysates is typically moderate, and DIA analyses often fail to reproducibly identify, or to provide correct quantitative information for, a large fraction of lysosomal proteins. An approach that enables the direct analysis of all lysosomal proteins in highly complex samples with excellent performance is targeted MS, such as parallel reaction monitoring (PRM) [63, 64]. This is especially important for sample types for which lysosome enrichment is not possible, such as body fluids, human tissues, or frozen specimens.

Taken together, these diverse approaches provide spatial information and increase the analytical performance for low‐abundant lysosomal proteins, enabling the investigation of lysosomal biogenesis. While the analysis of M6P‐modified lysosomal proteins is limited to a subgroup of the lysosomal proteome (mainly luminal), it does not require genetic manipulation or any type of modification procedure. Proximity biotinylation enables the labeling of (almost) all lysosomal membrane and associated proteins and their interactors but does require the exogenous expression of either BirA*/TurboID or APEX/APEX2. While APEX is smaller and catalyzes the labeling reaction with minimal incubation periods (∼1 min), it requires the addition of H_2_O_2_, which can be toxic for cells. Conversely, BirA* is bigger and depends on longer incubation periods but only requires soluble biotin for labeling. Moreover, different variants of this enzyme have decreased reaction times (∼10 min for TurboID) and reduced size (miniTurbo). Similarly, cross‐linking allows for the labeling of a great variety of interacting proteins without the need for genetic manipulation but comes at a low sensitivity and large amounts of input material are needed.

## 2 Investigation of Lysosomal Biogenesis

**FIGURE 3:**
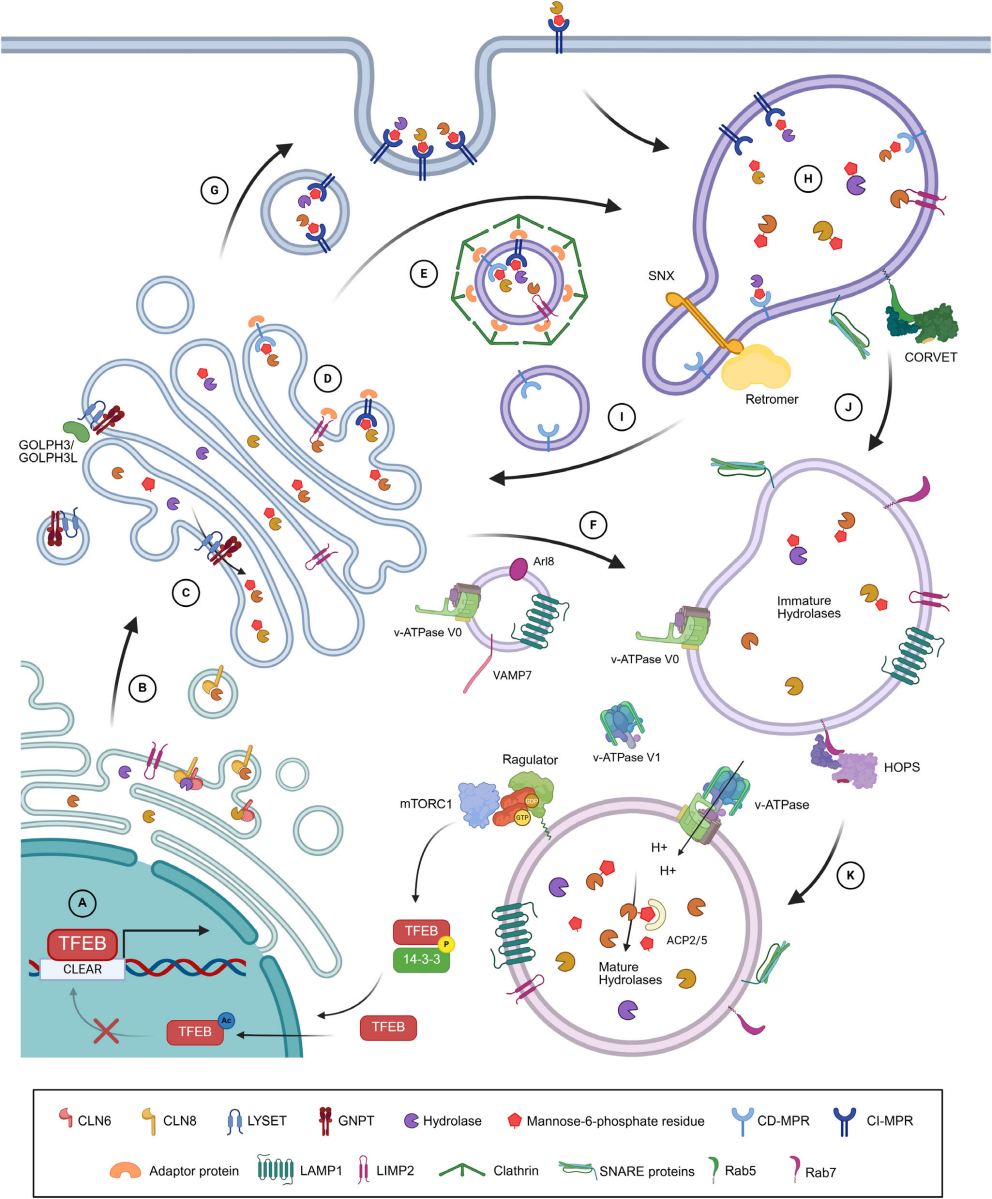
Lysosomal biogenesis and maturation. (A) TFEB binds to the coordinated lysosomal expression and regulation (CLEAR) elements for the regulation of expression of lysosomal, lysosome‐related, or autophagic genes. (B) Lysosomal proteins are synthetized in the endoplasmic reticulum (ER), lysosomal luminal proteins are transported by the CLN6‐CLN8 complex to the Golgi apparatus, while lysosomal membrane proteins do not require specific transport proteins. (C) In the Golgi, lysosomal luminal proteins are modified by the GNPT complex with mannose‐6‐phosphate (M6P), whose *cis*‐Golgi localization is regulated by LYSET and the COPI adaptors GOLPH3/GOLPH3L. (D) M6P‐tagged proteins are recognized by two receptors located in the *trans*‐Golgi network (TGN) and the cation‐dependent (CD) and cation‐independent (CI) M6P receptors (MPRs). (E) M6P‐modified hydrolases, together with lysosomal membrane proteins, are loaded in clathrin‐coated vesicles that fuse later with early endosomes. (F) Membrane proteins can also be delivered directly to late endosomes through LAMP carriers. (G) Secreted M6P‐modified hydrolases are recognized by the CI‐MPR, resulting in their receptor‐mediated endocytosis. (H) M6P‐modified lysosomal hydrolases converge in early endosomes and, due to the acidic environment of these vesicles, dissociate from MPRs. (J) Early endosomes fuse with late endosomes through a class C core vacuole/endosome tethering complex (CORVET)‐mediated mechanism. (I) The recycling of MPRs back to the TGN is mediated by the retromer complex. (K) Late endosomes mature into terminal lysosomes, where phosphatases eliminate the M6P modification from hydrolases. In the lysosomal membrane, nutrient sensing proteins such as mTORC1 are recruited for the control of lysosomal biogenesis by the phosphorylation of TFEB.

Lysosomal biogenesis is a multi‐step process, which requires the concerted action of several subcellular compartments. Following the transcriptional regulation of lysosomal gene expression in response to cellular metabolism, lysosomal proteins are synthesized at the endoplasmic reticulum (ER), where they are glycosylated and transferred to the Golgi apparatus. From there, lysosomal proteins are trafficked mainly by intracellular vesicles to their final destination, the endolysosomal compartment, based on protein complexes recognizing specific sorting motifs and post‐translational modifications (PTMs). Fusion of these vesicles with early endosomes presents the first step in the formation of new lysosomes, which further includes the maturation of a variety of lysosomal proteins by proteolytic processing and their acidification by the v‐ATPase complex to form fully catalytically active terminal lysosomes (Figure 3).

### 2.1 Lysosomal Signaling and Transcriptional Regulation of the Lysosomal Proteome

The lysosome is not only essential for the degradation of macromolecules but also fulfills a crucial role for the regulation of metabolic signaling. Two kinases that are known to be major players in the regulation of cellular growth and metabolism, mTORC1 and AMPK, were shown to be activated at the lysosomal surface, facilitating coordination between nutrient availability and cellular signaling [12, 13]. The best‐studied kinase in this context is mTORC1, whose activity is regulated by lysosomal/cytosolic nutrient levels and environmental cues [1, 2]. MS‐based phosphoproteomics has been crucial to identify mTORC1 substrates, both in large‐ and small‐scale experiments. Phosphoproteomic profiling of mTORC1 inhibition by Torin1 and rapamycin identified 127 phosphorylation sites on 93 proteins to be Torin1‐sensitive, of which 23 phosphorylation sites in 17 proteins were also rapamycin‐sensitive, uncovering a large network of putative mTORC1 substrates [65]. A major function of mTORC1 is the regulation of protein translation, for which crucial phosphosites were identified by co‐IP and MS analysis on several proteins, that are frequently utilized as readout for mTORC1 activity [66, 67].

Altogether, at least 117 mTORC1 substrate sites on 56 direct protein substrates are known to date, and were shown to be involved in a variety of cellular biosynthetic processes, such as autophagy, lipid metabolism, mitochondrial fusion, and ribosome biogenesis (reviewed by Battaglioni, et al.) [68]. The activity of mTORC1 is regulated both through upstream signaling pathways, whose state is transferred via phosphorylation of the tuberous sclerosis complex (TSC), a target of several kinases such as AMPK and AKT [69, 70, 71, 72, 73], as well as nutrient levels, which are sensed either through lysosomal transmembrane proteins, for instance, the amino acid transporter SLC38A9 [74] or the cholesterol transporter NPC1 [75], as well as cytosolic lysosome‐associated complexes [73, 76, 77, 78, 79, 80, 81]. These mechanisms allow the cell to adjust biosynthetic processes to nutrient availability, providing a switch between anabolic processes, such as protein translation, and catabolic mechanisms like the initiation of autophagy for salvage of intracellular components.

Importantly, a direct feedback loop between the activity of mTORC1 and lysosomal biogenesis exists, through regulation of the nuclear translocation of the MiTF/TFE family of basic helix‐loop‐helix leucine zipper transcription factors by their PTMs. Also, other important signaling pathways (ERK/MAPK, PKC, or AMPK signaling) impinge on this family of transcription factors, enabling regulation of lysosomal gene expression through multiple mechanisms [82]. The MiTF/TFE family of transcription factors includes MITF, TFEB, TFEC, and TFE3, which can form homo‐ or heterodimers that recognize a palindromic 10 bp DNA motif termed “coordinated lysosomal expression and regulation (CLEAR)” element [1, 83]. The activity of these transcription factors provides a direct feedback loop between the availability of nutrients and the modulation of lysosomal biogenesis [73, 84], numbers, size [83], catabolic activity [16, 85], and positioning [86, 87], as well as the expression of genes related to the autophagic machinery [84, 88]. The most thoroughly investigated protein in this context is TFEB, which is frequently also referred to as the “master regulator of lysosomal biogenesis” [1, 84] (Figure 3A). It was shown that nuclear translocation of TFEB occurs in response to various stimuli related to lysosomal activity, such as amino acid starvation [84], phagocytosis [6], oxidative stress [89], mitochondrial damage [90], inflammation [91], and physical exercise [92]; and that it is (almost) exclusively regulated by PTMs [82, 93].

MS‐based proteomics, typically in combination with TFEB IP [94] or in vitro kinase assays [95], stable isotope labeling of amino acids in cell culture (SILAC) [96], and/or phosphopeptide enrichment [94], played a crucial role in the investigation of TFEB PTMs, identifying mainly Ser/Thr phosphorylation sites as decisive factors. In this context, phosphorylation of TFEB by ERK2 [84], mTORC1 [96], PKC [95], GSK3β [95], and AMPK [94] at both unique and redundant sites was shown to be critical for the regulation of TFEB subcellular localization, leading to over 16 phosphorylation sites with known function to date [82, 97]. Phosphorylation of TFEB results in its cytosolic retention due to interaction with 14‐3‐3 proteins, thus inhibiting its nuclear translocation and transcriptional activity [88]. Besides phosphorylation, other PTMs were identified by MS‐based proteomic analysis of TFEB and shown to modulate its cellular localization and activity, including acetylation [98, 99, 100], ubiquitination [98], SUMOylation [101], glycosylation [102], and oxidation [103]. Additionally, it was shown that TFEB poly‐ADP‐ribosylation (PARsylation) [104] regulates its nuclear translocation in response to Wnt signaling, but no MS analyses were utilized in this study. While TFEB transcriptional activity is typically monitored by qPCR analysis of its target genes [84], also DIA‐LC‐MS/MS was utilized to characterize changes in TFEB‐induced lysosomal protein expression levels between WT and phosphorylation‐resistant TFEB versions in a cancer cell model [105].

Also, the modulation of lysosomal gene expression through PTMs was shown for proteins other than TFEB by proteomics approaches. For instance, while characterizing PKC signaling, it was shown that ZKSCAN3, a transcriptional repressor, translocated to the cytoplasm upon mTORC1 inhibition, suggesting a possible phosphorylation‐dependent regulatory mechanism of lysosomal gene expression. In follow‐up experiments, ZKSCAN3 phosphorylation at Thr153 was identified by LC‐MS/MS, and this site was assigned to JNK2 with in vitro kinase assays. The authors were further able to show that ZKSCAN3 phosphorylation drives its nuclear export, thereby inhibiting its transcriptional repressor function on lysosomal biogenesis [95], demonstrating phosphorylation‐dependent regulation of the transcriptional repression of lysosomal gene expression. In the context of a mouse model of lysosomal protease deficiency, STAT3‐dependent and TFEB‐independent regulation of lysosomal gene expression was demonstrated. This mechanism was identified through proteomic analysis of kidney lysosomes enriched by Percoll gradient centrifugation (Figure 2A) from stable isotope labeling in mammals (SILAM)‐labeled mice. The authors compared asparagine endopeptidase (AEP)‐deficient and WT animals by in‐gel digestion and LC‐MS/MS, revealing increased levels of lysosomal hydrolases in AEP‐deficient mice. This suggested a compensatory mechanism for the buildup of proteins caused by the AEP deficiency, and STAT3 was identified as a decisive transcription factor by bioinformatic analysis and biological follow‐up experiments [106].

Many cancer cells present with alterations of lysosomal and autophagic function, as well as changes in the expression levels of members of the MiTF/TFE family. To determine proteome‐wide changes occurring in cancer cells, whole cell lysates from two cancer‐derived induced pluripotent stem cell (iPSC) lines and their parental fibroblasts were investigated by tandem mass tag (TMT) labeling, peptide fractionation, and LC‐MS/MS. This revealed that cancer‐derived iPSCs exhibited high levels of MYC and the histone deacetylase 2 (HDAC2), but decreased levels of TFEB, compared to the parental fibroblasts. Through additional genetic and biological experiments, the authors suggested that MYC acts in opposition to TFEB, thus repressing lysosomal gene expression, potentially through an HDAC2‐dependent mechanism [107].

### 2.2 Lysosomal Protein Biosynthesis and Cellular Distribution

#### 2.2.1 Synthesis of Lysosomal Proteins at the ER and Sorting to the Golgi Apparatus

The majority of lysosomal proteins are synthesized at the ER, as they present either hydrolases, whose distribution and activity are strictly regulated, or transmembrane proteins that need to be co‐translationally inserted into membranes [3, 23]. Following the removal of the signal peptide, oligosaccharides are transferred onto asparagine residues within the sequence motif [N]X[S/T] [23]. Subsequently, they are delivered from the ER to the Golgi for further processing, from where they are targeted to their final destination, such as, endosomes or lysosomes, by various routes of vesicular transport [3, 108, 109]. For the characterization of these routes, MS‐based proteomics has provided invaluable insights.

The first known lysosome‐specific regulatory mechanism in this context takes place during the formation of COPII vesicles for anterograde ER to Golgi protein transport, where the two ER‐resident proteins CLN6 and CLN8 were shown to play crucial roles for lysosomal luminal proteins [110] (Figure 3B). For CLN6 knockout (KO) mice, so‐called “tritosomes” were induced by injection of animals with Triton WR‐1339. This compound accumulates in liver lysosomes, facilitating a shift in density, and hence their highly specific enrichment (Figure 2A). Lysosomal proteins for both WT and CLN6 KO tritosomes were quantified by TMT, identifying a total of 1691 proteins, of which 237 were lysosomal. LAMP2 and 10 hydrolases were downregulated in CLN6 KO compared to WT, while no significantly upregulated proteins were quantified [111]. For the investigation of CLN8 KO mice, liver lysosomes were enriched using Nycodenz density gradient centrifugation (Figure 2A). Comparative proteomic analysis was performed using label‐free quantification, where more than 100 lysosomal proteins were found to be reduced in their abundance relative to WT animals. Interestingly, a strong regulation was observed in the intensity levels of lysosomal luminal proteins, while lysosomal membrane proteins remained largely unaffected [112]. This indicated a specific transport mechanism for lysosomal luminal proteins (mainly hydrolases), while lysosomal membrane proteins are still correctly sorted analogously to other membrane proteins.

In a follow‐up study, this mechanism was further characterized, and it was shown that CLN6 and CLN8 form a complex, which the authors named “ER‐to‐Golgi Relaying of Enzymes of the Lysosomal System” (EGRESS). They demonstrated that EGRESS recruits lysosomal luminal proteins and facilitates their co‐loading with CLN8 into COPII vesicles, while CLN6 remains in the ER [110].

As both of these studies were performed in vivo, enrichment of lysosomes was restricted at the time to differential centrifugation. As this approach presents with poor recovery of lysosomal proteins [39], the recently introduced LysoTag mice, which enable Lyso‐IP from mouse tissues by overexpression of TMEM192‐3xHA, could yield better results [46].

#### 2.2.2 M6P Modification of Lysosomal Luminal Proteins in the Golgi Apparatus

Once newly synthesized lysosomal proteins reach the Golgi, they are processed, sorted, and packaged for their delivery to lysosomes [108]. Depending on the sub‐lysosomal localization of the respective lysosomal protein, distinct processing pathways occur [3, 108].

The vast majority of lysosomal luminal proteins undergo tagging with a phosphorylated mannose residue, which is connected to an N‐linked glycan moiety (M6P). This PTM facilitates their recognition by one of the two MPRs and their subsequent delivery to lysosomes [23]. The addition of M6P is catalyzed in the *cis*‐Golgi by the hexameric (α_2_β_2_γ_2_) N‐acetylglucosamine (GlcNAc)‐1‐phosphotransferase (GNPT) complex, whose three subunits are encoded by the genes *GNPTAB* and *GNPTG* [108] (Figure 3C). While the GNPTB and GNPTG subunits of the complex were identified by genetic approaches [113, 114], the identification of GNPTA was achieved through a combination of Golgi membrane fractionation, affinity purification with a GNPTG‐matrix, and MALDI‐TOF‐MS [115]. An important process for the function of GNPT is the activation of the GNPTAB precursor through proteolytic cleavage by site‐1 protease (S1P) upon its arrival in the Golgi. This cleavage generates the two transmembrane subunits, GNPTA and GNPTB, essential for enabling the complex's catalytic activity [116, 117]. More recently, the role of the complex’ γ subunit for the M6P‐tagging of lysosomal luminal proteins was investigated by proteomic analysis of GNPTG KO cells. Using SILAC‐based quantification of GNPTG KO and WT cells, the authors compared SPIONs‐based lysosome‐enriched fractions (Figure 2B) [118]. Furthermore, cells were treated with NH_4_Cl, which lowers intracellular pH, resulting in the disassociation of M6P‐modified proteins from their receptors in the TGN and, consequentially, secretion to the cell culture medium. From the conditioned medium, the authors enriched M6P‐modified pre‐forms of lysosomal hydrolases with anti‐M6P single‐chain antibodies (Figure 2D). LC‐MS/MS analysis of these fractions identified a reduction of 29 and 11 hydrolases in lysosome‐enriched fractions and conditioned media, respectively, and hence their reduced modification by M6P [118]. While this identified an essential role of the γ subunit for ∼10 lysosomal luminal proteins, it also demonstrated that the complex remains functional without this subunit and is able to correctly phosphorylate the majority of lysosomal hydrolases.

The GNPT complex is anchored to cellular membranes via two transmembrane domains, and its correct localization in the polarized Golgi is crucial for its function [119, 120]. How the complex maintains its proper compartmentalization within the Golgi was unclear until recently, when three independent studies identified an essential role of the uncharacterized transmembrane protein 251 (TMEM251) in the M6P modification of lysosomal hydrolases, which was therefore renamed lysosomal enzyme trafficking factor (LYSET) and GNPTAB cleavage and activity factor (GCAF) [28, 121, 122]. LYSET was found to play an essential role in anchoring GNPT to the *cis‐*Golgi membrane, thereby facilitating its retention in the organelle (Figure 3C). After identification of a lysosome‐related function in genetic screens, all studies also used MS‐based proteomics analyses of LYSET KO cells to characterize LYSET's role in the trafficking of lysosomal luminal proteins. With both DIA and PRM, a reduction of 38 lysosomal luminal proteins in whole cell lysate samples and a concomitant increase of their levels in conditioned media were demonstrated. Furthermore, in one study, the characterization of M6P‐modified peptides by IMAC enrichment (Figure 2D) and MS analysis identified a lack of M6P in LYSET KO cells, as the underlying reason [28]. While LYSET‐deficient cells did not display decreased mRNA levels and pro‐enzyme forms for CTSB, CTSL, and HEXB, mature lysosomal hydrolases were significantly downregulated. Label‐free LC‐MS/MS analysis of lysosome‐enriched fractions (Figure 2B) displayed a reduction in lysosomal hydrolases and other luminal proteins that were subsequently detected in the secretome of LYSET‐deficient cells [122]. More recently, a study utilized DIA‐LC‐MS/MS analyses of whole cell proteome and secretome samples of KO cells for the COPI adaptors GOLPH3/GOLPH3L, which facilitate the selective retrieval of Golgi‐resident proteins to maintain their correct intra‐Golgi localization (Figure 3C). Intriguingly, GOLPH3/GOLPH3L KO cells showed a similar phenotype as LYSET KO cells. The authors were able to demonstrate that retention of LYSET in the Golgi is dependent on these proteins and that they are, hence, indispensable for the M6P modification of lysosomal proteins [123].

#### 2.2.3 Identification of Mannose‐6‐Phosphate‐Modified Lysosomal Luminal Proteins

To date, the M6P pathway presents the best‐characterized transport route for lysosomal proteins, as this modification allows for highly specific affinity enrichment. It has therefore been characterized through multiple experimental approaches using cells grown in culture, human samples, and rat/mouse tissues.

In early studies, M6P‐modified proteins were enriched from the conditioned media of NH_4_Cl‐treated U937 cells using affinity columns containing the immobilized soluble domain of the cation‐independent M6P receptor (sCI‐MPR). Eluate fractions were separated by SDS‐PAGE or 2‐dimensional electrophoresis (2‐DE), followed by in‐gel digestion and protein identification using MALDI‐MS/MS. This led to the identification of 15 proteins, including 12 known lysosomal hydrolases and three potential novel M6P‐modified proteins [124]. In a complementary study, the authors applied the same approach to U937 and MCF7 cells, resulting in the identification of 22 proteins, of which 16 were known lysosomal hydrolases, while the remaining proteins were described as potential novel lysosomal proteins [125]. In order to boost numbers of lysosomal proteins, also primary fibroblasts from double KO mice for both MPRs were investigated by a combined cation‐dependent (CD‐)/CI‐MPR affinity matrix. Eluate fractions were separated by 2‐DE, and individual gel spots were analyzed by peptide mass fingerprinting with MALDI‐TOF‐MS. This led to the identification of 34 known lysosomal proteins and four candidate proteins, for three of which the authors could demonstrate retention of overexpressed tagged versions on M6P affinity columns [126].

Subsequent investigations utilized a variety of human samples, including brain, plasma, and urine, to further identify (putative) lysosomal proteins. From homogenized human *post‐mortem* brain tissue, M6P‐modified proteins were enriched using sCI‐MPR affinity columns, separated using 2‐DE, and in‐gel digests of ∼800 spots were analyzed by peptide mass fingerprinting with MALDI‐TOF‐MS. Also, an unfractionated sample was in‐solution digested and analyzed with LC‐MS/MS. In total, 41 known lysosomal proteins were found, including 11 that were not previously described to be M6P‐modified, and 9 uncharacterized proteins were identified that had not yet been linked with lysosomes [127]. Following a similar strategy, the presence of mis‐sorted M6P‐modified proteins in plasma samples of healthy individuals was investigated. Following an M6P‐double enrichment strategy, proteins were separated using SDS‐PAGE and fractionated into 30 slices, as well as by 2‐DE, from which 60 spots were excised. All samples were in‐gel digested and analyzed by LC‐MS/MS, resulting in the identification of 37 known and 27 putative lysosomal proteins [128]. Finally, in‐solution digestion and nano‐LC‐MS/MS analysis of M6P‐affinity enriched proteins from urine samples identified 48 known and 9 potentially novel lysosomal proteins [129].

To further increase the numbers of identified M6P‐modified lysosomal proteins, rat and mouse tissues were investigated. For 17 different rat tissues, M6P‐containing proteins were affinity enriched by sCI‐MPR, in‐gel digested, and analyzed by LC‐MS/MS in combination with spectral counting. Out of 772 identified proteins, 60 were known to be lysosomal, and 52 were novel candidates for lysosomal localization, based on the similarity of their enrichment profile to known lysosomal proteins [130]. Finally, as M6P is rapidly removed in the lysosome by ACP2 and ACP5, strongly reducing levels of M6P‐containing proteins in WT cells/tissues, various tissues of ACP5 KO mice were used for M6P affinity enrichment. Eluate fractions of sCI‐MPR enrichment experiments were separated by SDS‐PAGE, in‐gel digested, and subsequently analyzed by LC‐MS/MS. In total, 65 known lysosomal proteins were identified, as well as 165 glycoproteins that potentially represent novel lysosomal proteins. In follow‐up experiments lysosomal localization was demonstrated by immunostaining for four of them [131].

While these studies typically focused on the identification of M6P‐modified proteins, they did not identify the exact sites of M6P modification. To address this, deglycosylation experiments from sCI‐MPR‐enriched human plasma as well as human and mouse brain samples were performed by treatment with the endoglycosidases Endo H and PNGase F. This treatment results in distinct mass shifts at the respective modified amino acids, which were identified by LC‐MS/MS analysis. From these data, the authors were able to pinpoint 42 and 99 M6P phosphorylation sites on 28 and 49 known lysosomal proteins in plasma [128] and brain [132] samples, respectively. In plasma samples, 44 proteins were previously not associated with lysosomes [128], together with 43 proteins in the human brain and 22 in the mouse brain [132], suggesting potential novel lysosomal proteins.

More recently, an approach was introduced based on the Fe^3+^‐IMAC enrichment of M6P‐modified peptides from in‐solution digested whole cell lysates. Analysis of glycopeptides with a combination of higher energy c‐trap dissociation (HCD), which results in an M6P‐specific fragment ion, and triggering of an electron‐transfer/higher‐energy collision dissociation (EThcD) scan by this ion, enabled the annotation of spectra derived from M6P‐modified peptides, allowing for their identification. Importantly, due to the soft fragmentation properties of EThcD, it also allowed for a detailed characterization of individual M6P side chains. Application of this approach to HeLa and CHO cells, including KO cells for the lysosomal phosphatases ACP2 and ACP5, allowed for the identification of 46 M6P sites in 35 proteins. Also, for ACP2/ACP5 double KO cells, the authors observed a marked increase (up to 20‐fold) in M6P‐peptide intensity, underlining the role of these proteins in the removal of M6P [51].

Compared to the previously published datasets, which all provided indirect evidence, this study presents for the first time clear experimental proof of the M6P modification of individual peptides. Therefore, the combination of this MS analysis approach with the previously utilized strategies for M6P‐modified protein enrichment promises to yield additional insights into the regulation of lysosomal protein transport by this PTM.

#### 2.2.4 Investigation of Individual Mannose‐6‐Phosphate Receptors

M6P groups on lysosomal proteins are recognized by two different MPRs: the CD‐ and CI‐MPR. They are located in the TGN, TGN‐derived intracellular vesicles, endosomes, and at the plasma membrane, facilitating the transport of lysosomal proteins to the endocytic pathway [23] (Figure 3D). While the majority of both MPRs are located in intracellular vesicles, a small amount can be found at the plasma membrane. So far, however, only the CI‐MPR was demonstrated to function in M6P‐dependent endocytosis events at the plasma membrane, enabling the retrieval of (unintentionally) secreted lysosomal luminal proteins [23]. While the CD‐MPR contains a single M6P receptor homologous (MRH) domain, the CI‐MPR contains 15 repetitive MRH units that exhibit different binding properties for phospho‐monoester and ‐diester glycans on lysosomal enzymes and also facilitate the binding of non‐glycosylated ligands, such as the insulin‐like growth factor II (IGFII) [108].

The presence of two distinct MPRs raises the question of whether they are functionally redundant or if each of them transports a distinct population of lysosomal proteins. This was addressed through proteomic analysis of serum from KO mouse models for either the CD‐ or the CI‐MPR, as a lack of each individual receptor leads to a hypersecretion of M6P‐modified proteins into the respective animals’ blood. Utilizing a two‐column system, consisting of a first column containing the CD‐MPR (as it was determined previously to be of lower M6P affinity), followed by a second column with the CI‐MPR, the authors purified M6P‐modified proteins from the pooled serum of 100 mice per genotype and investigated individual eluate fractions from each column. In a control experiment, only the CD‐MPR column was used, enabling the identification of potential contaminants [133]. Through application of a combination of quantitative approaches, consisting of LC‐MS/MS‐based label‐free quantification of in‐solution digested and SDS‐PAGE‐fractionated samples, as well as isobaric tags for relative and absolute quantification (iTRAQ)‐labeling of in‐solution digested samples and analysis by LC‐MALDI‐TOF/TOF, the authors investigated differences between both MPRs. They identified discrepancies in the level of several proteins between the two mouse lines, indicating variable affinities of individual proteins to the CD‐ and CI‐MPR. This included the known lysosomal proteins CREG1, TPP1, and HPSE (higher affinity for CD‐MPR) or MAN2B1, CTSD, PSAP, and GRN (higher affinity for CI‐MPR) [133]. As the observed differences were rather moderate, and animals lacking either CD‐MPR [134] or CI‐MPR [135] do not exhibit strong effects, while a combined deficiency results in a lysosomal storage phenotype [136, 137], these data draw a picture in which the two MPRs are partially redundant in their function with respect to M6P‐modified lysosomal proteins, while the CI‐MPR also fulfils additional roles. Further knowledge could be gained by the utilization of ETD‐based fragmentation techniques, as identification of the exact M6P modification sites would help to explain the functional differences in binding of individual proteins to the CI‐/CD‐MPR.

When these studies were performed, it was not clear if GRN was indeed a lysosomal protein and if it interacted directly with the CI‐MPR. This question was answered in a subsequent investigation utilizing SILAC‐labeling and LC‐MS/MS analysis of co‐IPs for GFP‐PGRN and FLAG‐PSAP. The authors identified a direct interaction of these two proteins and their binding to the CI‐MPR, implying a SORT1‐independent trafficking pathway for GRN, which they confirmed by molecular biology experiments [138].

In addition to well‐characterized players in lysosomal biogenesis, other lysosomal proteins exist that fulfill important functions, as their loss/mutation results in severe pathological conditions, but whose mode of action remains poorly understood. One of these proteins is the lysosomal multi‐pass transmembrane protein CLN3, mutations of which cause the fatal neurodegenerative LSD Batten disease. Two independent proteomics studies connect CLN3 to the CI‐MPR, and hence lysosomal enzyme trafficking. In the first study, SILAC‐labeling and LC‐MS/MS analysis were used to compare SPIONs‐enriched lysosomal fractions (Figure 2B) of cerebellar cells derived from WT and CLN3‐defective (Cln3^Δex7/8^) mice. Out of 70 lysosomal proteins, 28 hydrolases showed significantly reduced levels in CLN3‐defective cells. Controversially, increased levels of the CI‐MPR were detected, suggesting an impairment in M6P‐modified lysosomal enzyme trafficking and/or uptake due to loss of CLN3 [139]. More recently, another study characterized the role of CLN3 in ARPE19 KO cells through DDA‐LC‐MS/MS‐based label‐free quantification of CLN3 co‐IPs under different metabolic conditions. The authors identified 107 and 158 interactors under fed and starved conditions, respectively, of which 88 were shared between both. The latter included protein complexes such as BORC, SNARE, and retromer, which play important roles in lysosomal motility, fusion with other vesicles, and endosome‐to‐Golgi retrieval of receptors. Interestingly, the CI‐MPR was among the most enriched CLN3 interactors, and the authors observed alterations of its subcellular localization in CLN3 KO cells, for which they performed further follow‐up experiments. This demonstrated that lack of CLN3 results in the lysosomal accumulation and hence degradation of the CI‐MPR, as well as its mis‐trafficking to the plasma membrane and accumulation in early endosomes. Also, in these cells LC‐MS/MS analysis of Lyso‐IP‐enriched lysosomes was performed (Figure 2C), identifying a reduction in the levels of 14 hydrolases, which were identified to be mis‐sorted to the secretory pathway, probably due to a lack of binding to the CI‐MPR. Taken together, these results indicate a crucial role of CLN3 in the delivery of lysosomal hydrolases through regulation of CI‐MPR trafficking, further confirming the relationship between these two proteins [140]. Furthermore, the recently introduced LysoTag mice were used to study the impact of CLN3 loss on the lysosomal metabolome in vivo through enrichment of lysosomes from brain and liver. The authors identified accumulation of glycerophosphodiesters (GPDs), together with an overall altered phospholipid catabolism, and, through subsequent in vitro experiments, suggested that CLN3 acts as a lysosomal exporter of GPDs or participates in their transport [46].

#### 2.2.5 M6P‐Independent Trafficking of Lysosomal Luminal Proteins

Not all lysosomal luminal proteins rely for their trafficking on M6P, and several alternative mechanisms have been identified with the help of proteomics approaches. The LC‐MS/MS analysis of SPIONs‐based lysosome‐enriched fractions (Figure 2B) from differentially SILAC‐labeled primary fibroblasts of GNPTAB KO and WT mice demonstrated that 20% of lysosomal hydrolases, including CTSD and CTSB, were not affected by the loss of M6P [141]. Follow‐up studies on this finding further revealed that SORT1, which was shown before to play a role in the trafficking of lysosomal proteins, such as CTSD, CTSH, ASM, and PSAP [142, 143], was not solely required for the trafficking of CTSD and CTSB. The latter was also carried out by members of the LDLR family [141]. In a subsequent investigation, the same authors analyzed tritosomes (Figure 2A) from GNPTAB KO/WT mice by in‐solution digestion and label‐free quantification with LC‐MS^E^, covering 67 known soluble lysosomal proteins. Their data from these analyses showed that in fact only a small number of lysosomal proteins (NEU1, CTSF, CTSL, and NPC2) relied on M6P for their sorting, while sufficient amounts of the majority of the other lysosomal proteins reached their final destination by alternative M6P‐independent trafficking routes [144]. As these findings contradict their previous analyses, as well as the more recent works concerning GNPT [118], LYSET [28, 121, 122], and GOLPH3/GOLPH3L [123], it could be due to specific conditions in liver cells in situ or the use of tritosomes, as most other experiments were performed in cell culture. Additionally, the authors utilized a distinct MS analysis strategy (LC‐MS^E^), opposite to all other proteomics studies, which may affect the results.

Also, individual protein‐specific M6P‐independent trafficking routes exist, such as the delivery of the lysosomal hydrolase GBA by the membrane protein LIMP2, which was initially identified through LC‐MS/MS analysis of LIMP2 co‐IPs [145] (Figure 3D).

#### 2.2.6 Transport and Sorting of Lysosomal Membrane Proteins

Lysosomal membrane proteins are transferred from the ER to the Golgi membrane by COPII vesicles [146]. From there, they are further packaged into specific transport vesicles destined for the endolysosomal system, not based on M6P modification, but on sorting signals in their cytosolic tail region (Figure 3D). Lysosomal sorting motifs typically exhibit either a YXXØ or [D/E]XXXL[L/I] motif, with certain additional features that affect the functionality for lysosomal delivery, such as the location relative to the transmembrane domain, the presence of glycine or other amino acids with small side chains before tyrosine residues, or an acidic amino acid at the [D/E] position [23]. Besides these sorting signals, the trafficking of certain membrane proteins is mediated via PTMs, such as the N‐glycosylation of TMEM106B or the prenylation of CLN3 [147].

Recently, the trafficking of LAMP1, one of the most abundant lysosomal membrane proteins, was investigated through a combination of the Retention Using Selective Hooks (RUSH) system, proximity biotinylation by APEX2 (Figure 2E), and MS‐based proteomics in polarized neurons throughout the biosynthetic pathway. With their approach termed “Protein Origin, Trafficking, And Targeting to Organelle Mapping” (POTATOMap), the authors characterized the LAMP1 interactome at three different timepoints (20 min, 1 h, and 4 h) after ER release, through biotin enrichment, on‐bead digestion, and label‐free quantification by DDA‐LC‐MS/MS analysis. In total, they identified 580 putative members of the biosynthetic LAMP1 interactome (65/387/276 proteins at 20 min/1 h/4 h). Proteins enriched at 20 min were predominantly related to the early secretory pathway, including the ER and cis‐Golgi (e.g., SEC24A, SEC24B, COPA, COPB1, and COPB2); interactors at 1 h were related to the TGN, post‐Golgi vesicular trafficking, and endosome transport (e.g,. ARF1, ARF5, GGA1, TBC1D30, and VPS35); and those enriched at 4 h were mainly related to lysosomes, metabolic signaling, and synaptic activity (e.g., ARL8, RUFY3, TMEM106, SYT4, SYT5, and SLC12A5). The latter also included typical membrane proteins located at terminal lysosomes, such as mTOR, LAMTOR1, LAMTOR3, RAGA, RAGC, MCOLN1, and NPC1. Based on these data, the authors drew a picture of LAMP1's role in the vesicle‐based replenishing of terminal lysosomes in axons and proposed possible lysosome‐related trafficking mechanisms through the axon [58].

The combination of the RUSH system with proximity biotinylation presents an ideal approach for such studies. While TurboID could be used instead of APEX, the required longer labeling times (typically 10 min) of TurboID would present a broader distribution of time‐specific interactors. Alternatively, the interactome of individual proteins could also be investigated by the RUSH system and co‐IP‐MS experiments or IP from individual subcellular fractions such as ER, Golgi, or endosomes/lysosomes.

### 2.3 Sorting of Lysosomal Proteins Into Trafficking Vesicles

The sorting of lysosomal proteins for their subsequent delivery occurs in the TGN [148]. Key determinants for their segregation are signals present in the cytosolic domain of lysosomal membrane proteins and the recognition of M6P on luminal proteins by MPRs, which are recognized by adaptor proteins (APs). APs (AP‐1‐5) then capture their specific cargo and recruit scaffolding proteins, initiating the budding of coated‐transport vesicles [149]. The best‐characterized sorting process is the MPR‐dependent sorting of luminal proteins, where the binding of AP‐1 to MPR recruits the Golgi‐localized γ‐ear‐containing Arf‐binding family of proteins (GGAs), which act as clathrin adaptors [150] (Figure 3E). To further characterize individual functions of AP‐1 and GGAs, clathrin‐coated vesicles (CCVs) were enriched by Percoll density centrifugation experiments (Figure 2A) from differentially SILAC‐labeled cells in which AP‐1 or GGA2 was rerouted to mitochondria and investigated by LC‐MS/MS. Out of the 99 and 29 proteins that presented with reduced levels due to the rerouting of AP‐1 and GGA2, respectively, various vesicle coat components were identified for AP‐1, including GGA2 itself as well as eight SNAREs, indicating overlapping functions [151]. More recently, an alternative route for the delivery of lysosomal membrane proteins was described, so‐called LAMP carriers, which are small uncoated vesicles positive of LAMP1/LAMP2 and devoid of AP‐2, TGN46, and CI‐MPR (Figure 3F). It was observed that these vesicles fuse directly with late endosomes through recruitment of ARL8B, VPS41, and VAMP7, and it was suggested that they can transport NPC1 and the v‐ATPase V0 domain, which are localized in the lysosomal membrane [152, 153].

For future experiments with the goal of the unbiased characterization of LAMP carriers using MS‐based approaches, either proximity biotinylation experiments through LAMP fusion proteins could be utilized, or organelle immunoprecitations (IPs) could be carried out either through the expression of tagged LAMP versions or high‐affinity experiments against the endogenous proteins.

## 3 Investigation of Lysosome Maturation

The *de novo* generation of lysosomes starts with vesicles derived from the endocytic pathway, that is, endosomes, which receive lysosomal proteins from the various transport routes outlined above in a gradual process. During this transition, characterized by the addition of both lysosomal luminal and membrane proteins, early endosomes undergo a series of dynamic changes in both protein and lipid composition, transforming through late endosomes towards fully mature and catalytically active lysosomes. In addition to the different intracellular pathways for the direct trafficking of lysosomal luminal proteins, they can also reach it through a detour encompassing the secretory pathway and CI‐MPR‐dependent receptor‐mediated endocytosis at the plasma membrane [148] (Figure 3G). For both the intracellular‐ and endocytosis‐related transport pathways, MS‐based proteomics approaches have provided invaluable insights for the identification of their respective cargo.

The cargo of MPR‐CCVs was investigated through sucrose density gradient centrifugation‐based CCV enrichment from HeLa cells (Figure 2A) for siRNA‐mediated knockdown of the clathrin heavy chain, resulting in clathrin‐devoid vesicles. Through in‐solution digestion, iTRAQ‐labeling, peptide fractionation, and LC‐MS/MS analysis of CCV‐enriched fractions, 552 proteins were quantified, of which 63 were identified as putative CCV proteome, due to their higher abundance in WT conditions relative to clathrin heavy chain knockdown. This included several AP‐1/AP‐2 complex subunits, other adaptor and transport machinery‐related components, and known cargo proteins such as lysosomal hydrolases. An additional 28 proteins were identified that were not previously associated with CCVs, such as the post‐Golgi SNARE proteins STX6, STX7, and STX8, as well as AP‐3, retromer, and several subunits of the BLOC‐1 complex, which is involved in lysosomal transport [154].

During lysosomal biogenesis, the v‐ATPase complex is incorporated in the endosomal membrane, leading to their acidification. The resulting pH change correlates with lysosomal maturity and hydrolytic activity, with the lowest pH values in terminal lysosomes. In the mildly acidic environment of (late) endosomes, lysosomal hydrolases dissociate from the MPRs and are released to the endosomal lumen (Figure 3H), while MPRs are recycled to the TGN through specialized vesicles [3] (Figure 3I). The currently best‐characterized player in this context is the multi‐subunit retromer complex, consisting of sorting nexins (SNXs) and a VPS26/VPS29/VPS35 trimer [155, 156], whose compounds have been investigated in several proteomics studies. Through expression of several GFP‐labeled members of retromer (CI‐MPR, SNX1, SNX2, SNX5, SNX6, and SNX32) and SILAC‐co‐IPs in combination with in‐gel digestion and LC‐MS/MS analysis, their interactome was investigated in two independent studies. While the affinity purification of GFP‐CI‐MPR identified all four known retromer‐associated SNX proteins as well as SNX27 as high‐confidence interactors, the CI‐MPR was detected in IPs of SNX5, SNX6, and SNX32. Through a variety of approaches, including gene editing, immunofluorescence microscopy, and co‐IPs, both studies demonstrated that CI‐MPR recycling to the TGN is mediated by the BAR domain of SNX proteins through its direct interaction with the cytosolic tail of the CI‐MPR [157, 158].

Also, the VPS trimer's role in the trafficking of lysosomal proteins was investigated using Lyso‐IP (Figure 2C), TMT‐labeling, and LC‐MS/MS analysis of VPS35 KO cells. The authors identified 246 proteins to be reduced in VPS35 KO lysosomes, including all components of the BORC and Ragulator complex, ARL8, RAGA/C, and various lysosomal hydrolases. The underlying mechanisms were further investigated by cell surface biotinylation and LC‐MS/MS, revealing a widespread mis‐localization of known retromer cargo proteins at the cell surface and the lysosome of VPS35 KO cells. While, among others, the known cell surface proteins GLUT1, NOTCH1, and NOTCH2 were enriched in lysosomes, the endosomal/lysosomal proteins LAMP1, LAMP2, CLN3, and CI‐MPR were located in the plasma membrane, indicating a critical role for VPS35 in the trafficking of lysosomal proteins [159].

Once soluble lysosomal proteins reach their final destination, M6P moieties are removed by the acidic phosphatases ACP2 and ACP5 [160] (Figure 3K). Their substrate specificity was investigated in ACP2, ACP5, and ACP2/ACP5 KO mice through the enrichment of tritosomes (Figure 2A) followed by in‐solution digestion/LC‐MS/MS and 2‐D PAGE/MALDI‐TOF‐MS analysis. In total, the authors identified 40 substrates of ACP2 and ACP5 and were able to show that ACP2 is dispensable for dephosphorylation of the majority of the M6P‐tagged proteins. Interestingly, for NPC2 an additive effect on its isoelectric point was discovered, suggesting additional functions for ACP2 and ACP5 on this protein [160].

## 4 Identification of Lysosomal Proteins By Proteomic Analysis of Mature Lysosomes

In the early days of lysosomal proteomics, enrichment of intact lysosomes was mainly conducted with a focus on membrane proteins by differential centrifugation approaches (Figure 2A) as orthogonal approach to various studies focusing on the M6P‐dependent identification of lysosomal luminal proteins. Utilizing tritosome enrichment from rat livers (Figure 2A), membrane fraction generation, ion‐exchange chromatography fractionation, and analysis by SDS‐PAGE and LC‐MS/MS, a study identified 215 proteins as putative lysosomal integral membrane proteins, including several known members [161]. To add another layer of information, comparison of tritosome induced and control samples by iTRAQ 8‐plex labeling was performed with LC‐MS/MS and MALDI‐MS/MS to monitor changes in protein distribution between density gradient centrifugation fractions and hence lysosomal localization. This enabled the identification of 545 proteins, of which 38 were assigned as potentially lysosomal based on their migration pattern [35]. In a subsequent study, lysosomes were enriched from human placenta using a combination of Percoll, sucrose, and iodixanol density gradient centrifugation and disrupted by treatment with methionine methyl esters (Figure 2A). The resulting membrane fraction was separated using SDS‐PAGE, in‐gel digested, and analyzed by LC‐MS/MS, resulting in the identification of 58 known lysosomal membrane proteins, including 17 associated with the v‐ATPase complex. Also, 86 proteins were enriched, of which 12 presented novel proteins with unknown function. For two of the candidates, TMEM192 and CCZ1, lysosomal localization was confirmed by co‐immunostaining with LAMP2 [162]. Lastly, using a discontinuous Nycodenz density gradient (Figure 2A), hypoosmotic shock treatment, Triton X‐114‐phase separation, and ultracentrifugation, followed by LC‐MS/MS analysis of individual fractions, lysosomal membranes were investigated. Based on a spectral counting‐based quantification and bioinformatic analysis, 46 proteins were determined as potentially novel lysosomal proteins, and for nine of them colocalization with the lysosomal marker protein LAMP1 was demonstrated by fluorescence microscopy [163].

In more recent studies, typically the complete lysosomal proteome was investigated, which is also related to the capabilities of modern mass spectrometers to identify and quantify large numbers of proteins from single‐shot analyses. For instance, thorough characterization of SPION‐enriched lysosomes (Figure 2C) from mouse embryonic fibroblasts (MEFs) by our group with different sample preparation approaches resulted in the identification of more than 7000 proteins, raising the question, how many of these are truly lysosomal [164]. To enable differentiation between contaminants/background signals and lysosomal proteins, we included a differentially SILAC‐labeled population of control cells that did not receive SPIONs and pooled samples before lysosome enrichment. With this strategy, we investigated lysosome‐enriched fractions of six different immortalized cell lines (HEK293, HeLa, HuH‐7, SH‐SY5Y, MEF, and NIH3T3), observing for all of them two normally distributed populations, representing background signals and lysosomal proteins. Additional cross‐correlation analysis between enriched proteins of individual cell lines allowed to assign 89 potentially novel lysosomal proteins, for six of which we confirmed lysosomal localization by immunostaining [24]. As such identifications may still include non‐specifically binding proteins, we further utilized XL‐MS for the investigation of the lysosomal interactome, as it provides physical proof for interactions within a distance of < 35Å through the presence of a cross‐link. We treated both intact and ruptured SPION‐enriched lysosomes with the cross‐linker DSSO (Figure 2B,F), digested proteins, and enriched cross‐linked peptides with strong cation exchange (SCX) chromatography. LC‐MS/MS analysis of the resulting fractions identified 524 cross‐links for 111 lysosomal proteins, providing both structural information for known lysosomal proteins as well as novel putative interaction partners [27].

An approach that is, with respect to lysosomes, mainly utilized for the identification of lysosome‐associated proteins is proximity biotinylation (Figure 2E). As part of a major effort to characterize the general organelle interactome of a human cell, a total of 234 BirA* fusion proteins were generated across 32 distinct compartments of HEK cells, including seven lysosome‐localized baits (see above). After proximity biotinylation, avidin enrichment, on‐bead digestion, and LC‐MS/MS analysis, the authors identified 4424 prey proteins, of which they assigned 288 to lysosomes by spatial analysis of functional enrichment (SAFE), 173 by non‐negative matrix fractionation (NMF), and 145 by both methods. Subsequently, the lysosomal localization of seven potentially novel lysosomal proteins was confirmed by immunostaining [59]. Following another proximity biotinylation approach, APEX2 was fused to LAMP1 in order to characterize the LAMP1 interactome in iPSC‐derived neurons. Out of ∼130 identified proteins, ANXA11 was the highest rank interactor of LAMP1, and the authors showed in follow‐up experiments that this protein is a tether for RNA granules, enabling their axonal co‐transport with lysosomes [57].

Furthermore, individual protein complexes, such as the Ragulator, mTORC1, or v‐ATPase complex, were investigated in several studies using co‐IP experiments and LC‐MS/MS analysis, enabling the identification of novel complex members and interaction partners [85, 165].

### 4.1 Identification of Post‐Translational Modifications on Lysosomal Resident Proteins

The vast majority of lysosomal proteins are currently thought to be glycosylated at one point, and this modification plays a crucial role for lysosomal membrane proteins. Heavily glycosylated lysosomal membrane proteins (e.g., LAMPs or LIMPs) form the lysosomal glycocalyx, which lines the luminal face of the lysosomal membrane and prevents it from autodigestion by luminal hydrolases [166]. While information of protein‐specific glycan structures is missing, the N‐glycoproteome of the lysosomal glycocalyx was studied in CHO WT and NPC1 KO cells. Lysosomes were enriched using SPIONs (Figure 2B), glycans were released from their respective membrane proteins using PNGase F, and analyzed using HILIC‐UPLC and MALDI‐TOF/TOF‐MS, identifying the most abundant N‐glycan structures from the lysosomal glycocalyx and an altered N‐glycosylation pattern in the disease model [167].

While lysosomal proteins are regularly covered in unbiased large‐scale whole proteome PTM analyses, studies addressing the role of individual PTMs specifically for lysosomal function are scarce. To our knowledge, in addition to the abovementioned phosphoproteomics analyses with respect to mTORC1 and TFEB, only ubiquitination was addressed in a lysosomal context. Using LC‐MS/MS analysis of ubiquitination sites for LAMTOR1, the authors identified sites that were regulated by the deubiquitinase USP32. Through follow‐up experiments, the authors showed that ubiquitination of LAMTOR1 played a role in the interaction of Ragulator with the v‐ATPase complex, affecting mTORC1 recruitment and, therefore, its activity [168].

The investigation of PTMs on lysosomal proteins presents a promising avenue, ideally executed through the combination of enrichment procedures for lysosomes and individual PTMs. Due to the low abundance of both lysosomes and PTMs, this presented a challenging endeavor due to sensitivity issues in the past. The ever‐increasing speed and sensitivity of modern mass spectrometers promises to enable such experiments in the future.

## 5 Conclusion and Perspectives

Lysosomal biogenesis presents a multi‐step process involving several subcellular compartments and a variety of biological mechanisms. For each step of this process, MS‐based proteomics made important contributions, identifying novel proteins and regulatory mechanisms. This includes the regulation of lysosomal protein expression by PTMs, the transport of lysosomal proteins from the ER to the Golgi, their modification by M6P, the transport by Golgi‐derived vesicles to the endocytic system, the maturation of terminal lysosomes, and the characterization of their proteome. While certain pathways have been extensively characterized by MS, others were only addressed in very few studies, leaving room for new discoveries. Especially the continuously improving performance of modern mass spectrometers will be beneficial for such investigations, as lysosomal proteins are typically of low abundance. Particularly, with respect to the sorting and trafficking of lysosomal membrane proteins, the characterization of post‐Golgi carriers, and the implication of dysfunctional biogenesis and maturation in the context of lysosome‐related diseases, many open questions still remain. The currently available approaches for lysosome enrichment and the related proteomic toolkit present an ideal scenario to address these questions in future studies.

## Conflicts of Interest

The authors declare no conflicts of interest.

## Supporting information




**Supporting File**: pmic70058‐sup‐0001‐Table1.xlsx.

## Data Availability

The data that supports the findings of this study are available in the supplementary material of this article.
